# Spatial distribution and potential health risk of fluoride in drinking groundwater sources of Huaibei, Anhui Province

**DOI:** 10.1038/s41598-021-87699-6

**Published:** 2021-04-16

**Authors:** Yunhu Hu, Mu You, Guijian Liu, Zhongbing Dong

**Affiliations:** 1 School of Chemistry and Materials Engineering, Huainan Normal University, Huainan, 232001 China; 2 School of Bioology Engineering, Huainan Normal University, Huainan, 232001 China; 3 School of Earth and Space Sciences, University of Science and Technology of China, Hefei, 230026 China; 4 School of Earth and Environment, Anhui University of Science and Technology, Huainan, 232001 China

**Keywords:** Environmental sciences, Natural hazards

## Abstract

Fluoride enrichment in drinking groundwater at Huaibei leads to potential health risk to the residents. A total of 49 groundwater samples from groundwater sources were collected to evaluate the potential health risk of fluoride ingestion through drinking water for children and adults in Huaibei. Results shown that the average concentration of fluoride in centralized sources is less than that of decentralized sources, which may be attributed to different geological conditions including fluoride-rich minerals, environmental conditions and high fluoride waste discharge. The corresponding health risk value of fluoride in centralized source is lower than that in decentralized one, and the *HI* values of fluoride in the three exposed groups varied in the same order: infants > children > adults. Infants and children were more sensitive receptors to the non-carcinogenic health hazards of fluoride than adults. Special care should be taken to infants and children for the highly prone to health risk compared to adults.

## Introduction

Groundwater is one of the major sources of drinking water specifically in the arid and semi-arid regions on the earth^1,2^. Occurrence of fluoride in groundwater has attracted wide attention in the world due to it closely related to human health^3^. Fluoride is an essential micronutrient to maintain human bone development and growth^4^. Moderate fluoride intake can reduce the incidence rate of dental caries and promote bone development. Insufficient intake will cause incidence rate of dental caries^5^, while the excessive intake will cause endemic fluorosis^6,7^. Many studies show that fluoride content in drinking water is positively correlated with the incidence rate of fluorosis^8,9^. Long-term drinking of high fluoride water can cause maladjustment of calcium and phosphorus metabolism in human body, resulting in calcium deficiency and fluorosis^10^. Fluorosis can also make pregnant women postpartum paralysis^11^. Acute fluorosis can cause serious corrosion of stomach and degeneration of liver and kidney cells, fertility reduction including miscarriage, birth abnormalities and cancer in lungs, bone, bladder and uterus^10,12,13^. Studies have shown that developing children are more sensitive receptors than adults and face higher health risk of fluoride. High concentration of fluoride in drinking water have a negative effect on the children's intelligence quotient (IQ), and dental fluorosis may be the early indicator of children's IQ impairment^14,15^. In view of the above facts, it is of great practical significance to limit the intake of high fluoride for people's health. High fluoride groundwater health implication is a worldwide public health problem, especially in developing countries. It was reported that more than 60 million people are at high risk of fluorosis (dental and skeletal) in northwest China^16^. In order to ensure the safety of drinking water, the guideline fluoride value of 1.5 mg/L for drinking water was suggested by the World Health Organization^17–19^.

High concentration of fluoride in groundwater is mainly existed in the arid and semi-arid area with high evapotranspiration and low rainfall^1,20^. Elevated fluoride in groundwater is primary originated from lasting water–rock interactions and mineral weathering from fluoride bearing minerals such as fluorit, fluor apatite, biotite and phlogopite^9,21,22^. The concentration and transformation of fluorine in groundwater are determined by various factors, such as hydrogeochemical settings, temperature, pH, solubility of fluoride rich minerals and complexing ions^12,23,24^. In addition, high fluoride groundwater could be related to anthropogenic activities, such as phos-phate fertilizer application, burning of coal, glass and ceramic industry^25,26^. Meanwhile, fluoride could be released from fluoride-containing waste and waste residue by leaching and infiltration of rainfall and snowfall, finally resulted in potential soil and groundwater pollution. With the development of economy, the improvement of people's living standards and the strengthening of health awareness, more and more attention has been paid to the health problems related to drinking water. Because the fluoride in drinking groundwater has a direct impact on human health, it is necessary to study the distribution characteristics and influencing factors of fluoride in groundwater, and carry out health risk assessment on the residents, which is of great significance to ensure the safety of drinking water.

Huaibei is located in the arid and semi-arid area. Groundwater is the main water source to ensure its social and economic development^27^. For the high concentration of fluoride in the groundwater of Huaibei City (China), and the prevalence of dental fluorosis is more than 50–90%^28^. In recent years, the demand for water resources is increasing greatly with the accelerate development of socioeconomic and urbanization. The urban water supply of Huaibei is supplied by tap water. Due to the lack of water purification and other infrastructure in rural areas, some areas still use groundwater with high fluoride content as the main drinking and irrigation water source. Long-term excessive fluoride intake will affect the health of residents. Many studies had focused on the distribution characteristics and quality evaluation of groundwater fluoride in Wanbei Plain^27,29–31^. However, there is no comprehensive and systematic analysis on the distribution and causes of high fluoride water in Huaibei City, and there is no literature on the health risk assessment of fluoride in groundwater. Therefore, the spatial distribution characteristics, influencing factors analysis and health risk assessment of fluoride in Huaibei city provide the basis for the accurate evaluation of groundwater situation in Huaibei City, and have important practical significance for the prevention and control of fluoride disease and urban drinking water safety.

The groundwater system with typical representativeness and can be partitioned into the Quaternary aquifer and the Carboniferous limestone aquifer in Huaibei^27^. And groundwater plays an important role in economic and social development. The analysis of groundwater fluoride distribution characteristics and health risk assessment are of great significance to the protection of groundwater safety and residents' health. The main objectives of this study are to (1) determine the spatial distribution characteristics of fluorine; (2) analysis on influencing factors of fluoride in groundwater; (3) comparison of health risk assessment of fluoride in groundwater between infants, children and adults. The results from this study will provide scientific basis for managers to formulate health promotion strategies and measures, carry out fluorosis prevention and ensure groundwater drinking safety, so as to protect the health of residents.

## Materials and methods

### Study area

Huaibei is located in the north of Anhui Province with latitudes of 116° 23′–117° 02′ E and longitudes of 33° 16′–34° 14′ N, respectively. It governs three districts (Xiangshan District, Duji district and Lieshan District) and one county (Suixi County) with a total area of 2714 km^2^. Xiangshan mountain lies in the north of Huaibei with a height above sea level of 342 m. The terrain is flat, high in the northwest and low in the southeast. The quaternary sediments are mainly alluvial deposits, followed by alluvial proluvial and slope diluvial deposits. The thickness gradually thickens from north to south, ranging from 10 to 100 m. The main lithology is sub clay, clay, silty fine sand, etc. In the piedmont area, carbonate rocks are buried directly under the quaternary system. The annual average precipitation is 816.7 mm, of which the annual precipitation in July, August and September accounts for 70%.

The groundwater in Huaibei can be divided into karst water in carbonate aquifers and pore water distributed in loose Quaternary sediments. Karst water is mainly composed of Saiwu and Ordovician carbonatite. It is widely distributed and is the main water supply layer of Huaibei. The main recharge sources of groundwater are precipitation infiltration in exposed area of carbonate rock stratum in mountainous area and overflow recharge from overlying pore aquifer in plain area^31^. The main aquifer of pore water is composed of quaternary and neogene unconsolidated aquifer. The thickness is 40–120 m. The groundwater is mainly supplied by precipitation infiltration, and then by the river side. The groundwater resources are sufficient with water output of 100–2000 m^3^/day. The quality of shallow groundwater is generally worse than that of deep groundwater in loose Quaternary sediments, which is vulnerable to human activities such as industry and agriculture^27^. As a result, the types of shallow groundwater are complex and with high concentrations of NO_3_^−^ and total dissolved solids (TDS).

### Sample collection and test

**Figure 1:**
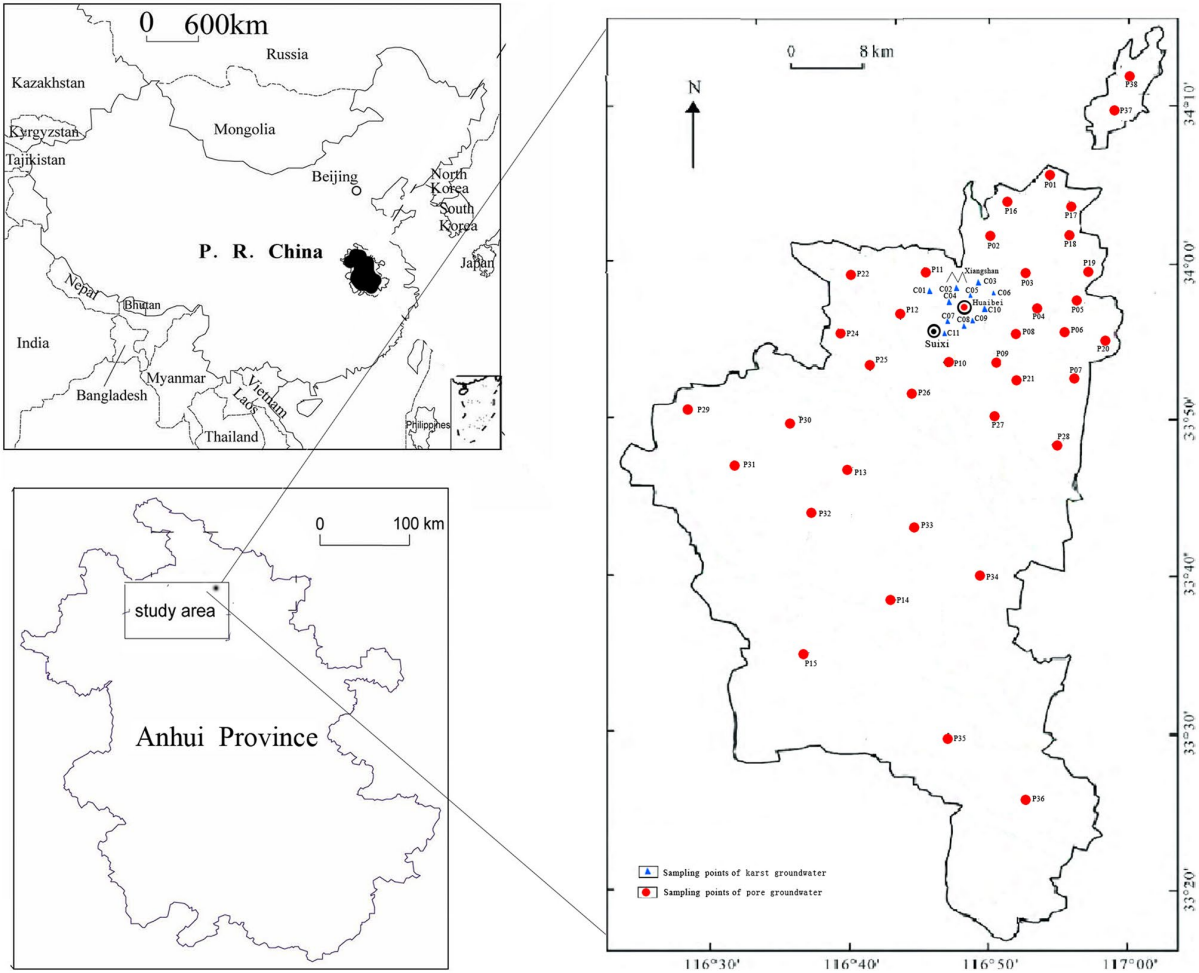
Location map and sampling points of study area (created with the software of Coreldraw X8).

A total of 49 groundwater samples were collected (Fig. 1), including 11 karst water samples and 38 pore water samples. The karst water samples were collected from centralized water supply wells in urban areas with the depth from 180 to 260 m, while the pore water samples were collected from decentralized groundwater sources in surrounding villages and towns or enterprises with the depth from 8 to 160 m. Each well was pumped for 10 min until about twice the volume of the well was discharged, and stable chemical conditions were achieved^2^. The sampling locations were recorded with a global positioning system (GPS) equipment (Garmin Etrex 10)^26^. Temperature, pH, EC, DO and TDS were measured with the water quality field kit (ESICO Model-1160E) in situ. The groundwater was sampled with plastic bottles after thoroughly rinsing with water from the well, acidified to pH 2 and stored at 4 °C in the refrigerator, all the samples were transported to the Laboratory of Anhui Geological Prospecting Bureau for detection as soon as possible. The concentrations of anions (F^−^ and NO_3_^−^) were measured by ion chromatography (Dionex, ICS-3000, USA), while cations (Ca^2+^, Mg^2+^, K^+^, Na^+^) were analyzed by atomic emission spectrometer (ICP-AES, Perkin Elmer Optima 5300DV, Waltham, Massachusetts, USA). The collection, storage and transportation of groundwater samples are carried out in accordance with the requirements of Regulation for water environmental monitoring (SL219-2013) set by Ministry of Water Resources P.R.C. The accuracy and precision of analyses were measured through spiked samples duplicate samples and analysis of the blanks, the relative standard deviation of determination for major ions measured major anions were within ± 5%.

### Health risk assessment model

Health risk assessment (HRA) is an effective probabilistic approach which links water pollution with human health^13,32^. It can quantitatively describe the harm of environmental pollution to human environment. As fluoride has a direct impact on human health^14^, the health risk assessment of fluoride in groundwater can provide a baseline information for ensuring the safety of drinking water and sustainable utilization of water resources. The health risk assessment model recommended by the United States Environmental Protection Agency (USEPA) has been widely used and confirmed in the water quality assessment of drinking water^7,33^. In this study, the model is used to evaluate the health risk of fluoride in groundwater.

Fluoride in drinking water mainly enters the human body through drinking water intake and skin contact. Many studies show that the value of risk through skin contact is very small and can be ignored relative to drinking water intake^1,13^. Since drinking water is the main risk source of fluoride to human bodies^11^, only drinking water intake is considered in this study. Fluoride is a non-carcinogenic risk factor, the health risk assessment of fluoride content in groundwater in the study area is carried out by using the non-carcinogenic risk assessment risk model. The calculation formula is as follows:

1 $${I}_{CDI}=\frac{C\cdot IR\cdot EF\cdot ED}{BW\cdot AT}$$

2 $$HI=\frac{{I}_{\mathrm{CDI}}}{RfD}$$

The formula parameters for the calculation of ${I}_{CDI}$ values are described in Table 1 obtained from previous studies^13,34–36^. Although adults are considered in the area, special attention should be pay to the children and infants. The studied people are classified into three groups: infants (less than 2 years), children (2 to < 16 years) and adults (≥ 16 years).

**Table 1:** Parameters employed for human health risk exposure assessment in groundwater.

Parameters	Unit		Children	Adults	Literature
C: Concentration of fluoride	mg/L	Fluoride concentration in this study			
IR: Ingestion rate of water (L/day)	L/day	0.5	1.5	2.4	^34^
EF: Exposure frequency	day/year	200	350	350	^36^
ED: exposure duration for risk assessment	year	1	12	64	^35^
BW: Average body weight	kg	6	20	65	^35^
AT: Averaging time	Day	200	4200	22,400	^13^
*RfD*: Oral reference dose	mg/kg day	0.06	0.06	0.06	^13^

Recommendations based on the standards of USEPA's health risk assessment, the threshold value of the non-carcinogenic risk index (*HI*) is 1. When *HI* > 1, it indicates that the non-carcinogenic risk of the human body exposed to the environment is large, and it poses a certain threat to the health of the human body; When *HI* < 1, it indicates that the risk is small and is regarded as acceptable.

## Results and discussion

### Distribution characteristic of fluoride

**Figure 2:**
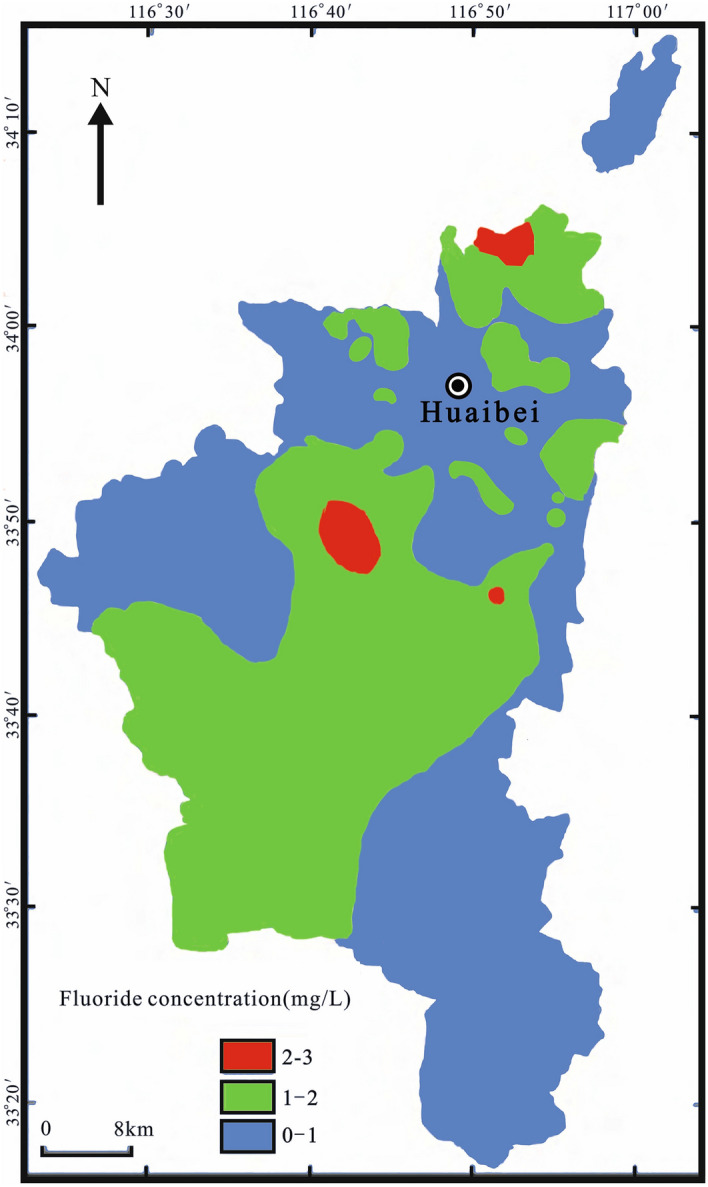
Map of fluoride concentrations in groundwater of study area (created with the software of ArcGIS desktop 10.7).

Table 2 indicated the parameters of groundwater, the parameters are compared to national^37^ and international standards^38^. The concentration of fluoride in centralized groundwater sources and decentralized groundwater sources ranging from 0.35 to 0.90 mg/L and 0.42 to 3.65 mg/L, with an average of 0.62 and 1.14 mg/L, respectively. The average concentration of fluoride in decentralized groundwater sources is higher than that of centralized groundwater sources. As shown in Fig. 2, the concentration of fluoride in centralized groundwater is less than the drinking water quality standard of fluoride, while 9 of 38 decentralized groundwater samples exceeded the drinking water quality standard of fluoride set by WHO, accounting for 23.68%.

**Table 2:** Descriptive statistics for groundwater samples in Huaibei, China.

Index	Karst water					Pore water					Chinese standard^37^	WHO Standard^38^
	Min	Max	Average	Standard deviation	Coefficient of variation	Min	Max	Average	Standard deviation	Coefficient of variation		
pH	7.14	7.34	7.58	0.16	0.02	7.36	8.37	7.91	0.27	0.03	6.5–8.5	6.5–8.5
K^+^	1.00	2.04	2.93	0.73	0.25	0.06	21.77	5.60	5.83	1.04	–	12
Na^+^	26.03	95.51	333.50	82.60	0.25	45.01	468.40	179.64	101.27	0.56	200	200
Ca^2+^	16.31	110.35	130.80	32.44	0.25	6.31	198.00	62.73	60.30	0.96	–	75
Mg^2+^	17.89	36.85	56.36	13.68	0.24	33.42	196.69	71.41	38.79	0.54	–	75
HCO_3_^−^	328.75	413.43	504.67	53.96	0.11	183.75	923.84	396.13	182.36	0.46	–	500
Cl^−^	29.80	68.45	169.42	39.43	0.23	31.55	420.44	110.04	92.89	0.84	250	200
SO_4_^2−^	63.08	185.15	272.70	58.80	0.22	33.75	1010.07	192.03	212.55	1.11	250	250
TDS	515.00	681.36	883.00	129.36	0.15	301.00	2791.19	890.76	527.89	0.59	1000	500
NO_3_^−^	8.80	24.24	45.89	15.27	0.33	0.00	25.97	9.92	7.95	0.80	20	50
F^−^	0.35	0.62	0.90	0.21	0.23	0.42	3.65	1.14	0.66	0.58	1.0	1.5

Concentration of ions and TDS is mg/L.

**Figure 3:**
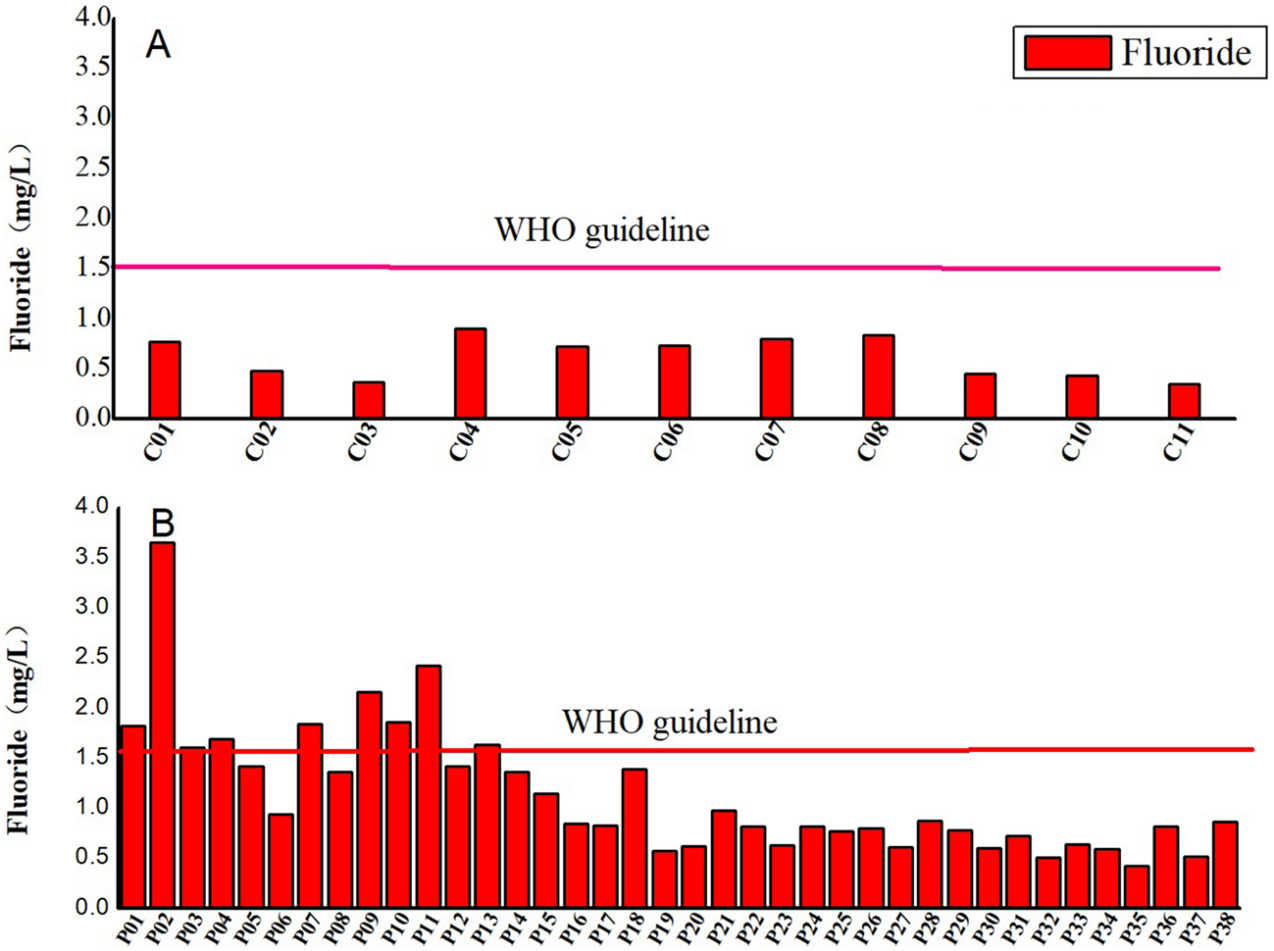
Comparison of mean fluoride concentrations in groundwater with WHO guideline of fluoride in drinking water: (**A**) Centralized groundwater sources. (**B**) Decentralized groundwater sources.

**Figure 4:**
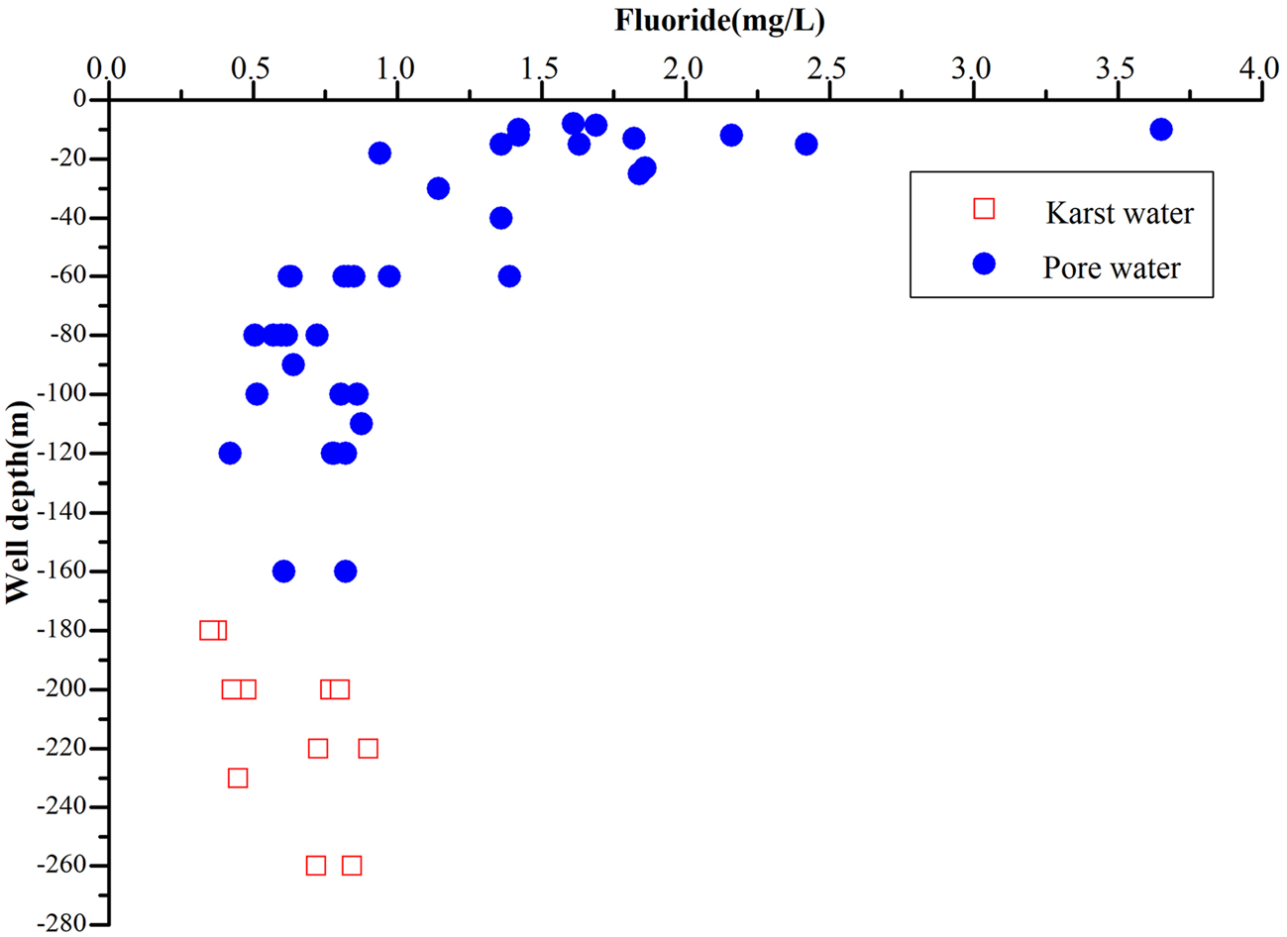
Relationship between fluoride concentrations and well depth.

There is no obvious regularity in the horizontal distribution of fluoride content (Figs. 2, 3), and the content is small in the northern Xiangshan karst area. The concentration of fluoride in groundwater increases from north to south, and the vertical distribution of fluoride in groundwater decreases with the increase of well depth (Fig. 4), which is closely related to the depth of water intake layer. The concentration of fluoride in shallow groundwater is higher than that in deep groundwater. Since the groundwater is the main source of industrial and agricultural production, it is necessary to evaluate the human health risk of groundwater in the study area.

### Sources of fluorine in groundwater

The enrichment of fluoride in groundwater is mainly related to the source of fluorine in rock and soil. Various fluoride minerals, including kaolinite, montmorillonite and hydromica, mica, apatite, tourmaline and hornblende are found in the aquifer formation of Huaibei^39^. Fluoride in fluorine minerals could be released with the rock-water interactions and lead to the increasing of fluoride in groundwater. The continuous dissolution of fluorine-containing minerals is the main resource of the enrichment of fluoride in groundwater, but its concentration variation also affected by many factors such as climate, topography and geology, lithologic structure, groundwater flow and environmental media. The shallow water is mainly vertical leaching of fluorine in vadose zone and leaching hydrolysis of fluorine in aquifer. The migration and enrichment of fluoride by horizontal runoff is relatively enhanced for the weakening of vertical alternation in deep water, which increases the fluoride content in aquifer. Meanwhile, drought climate and alkaline environment will lead to the increasing of fluoride content in groundwater. Some studies shown that the concentrated distribution area of high fluoride in groundwater is consistent with the high fluorine waste discharge sites such as coal mines and power plants^28^.

### Mutual relationships between fluoride and other ions

**Figure 5:**
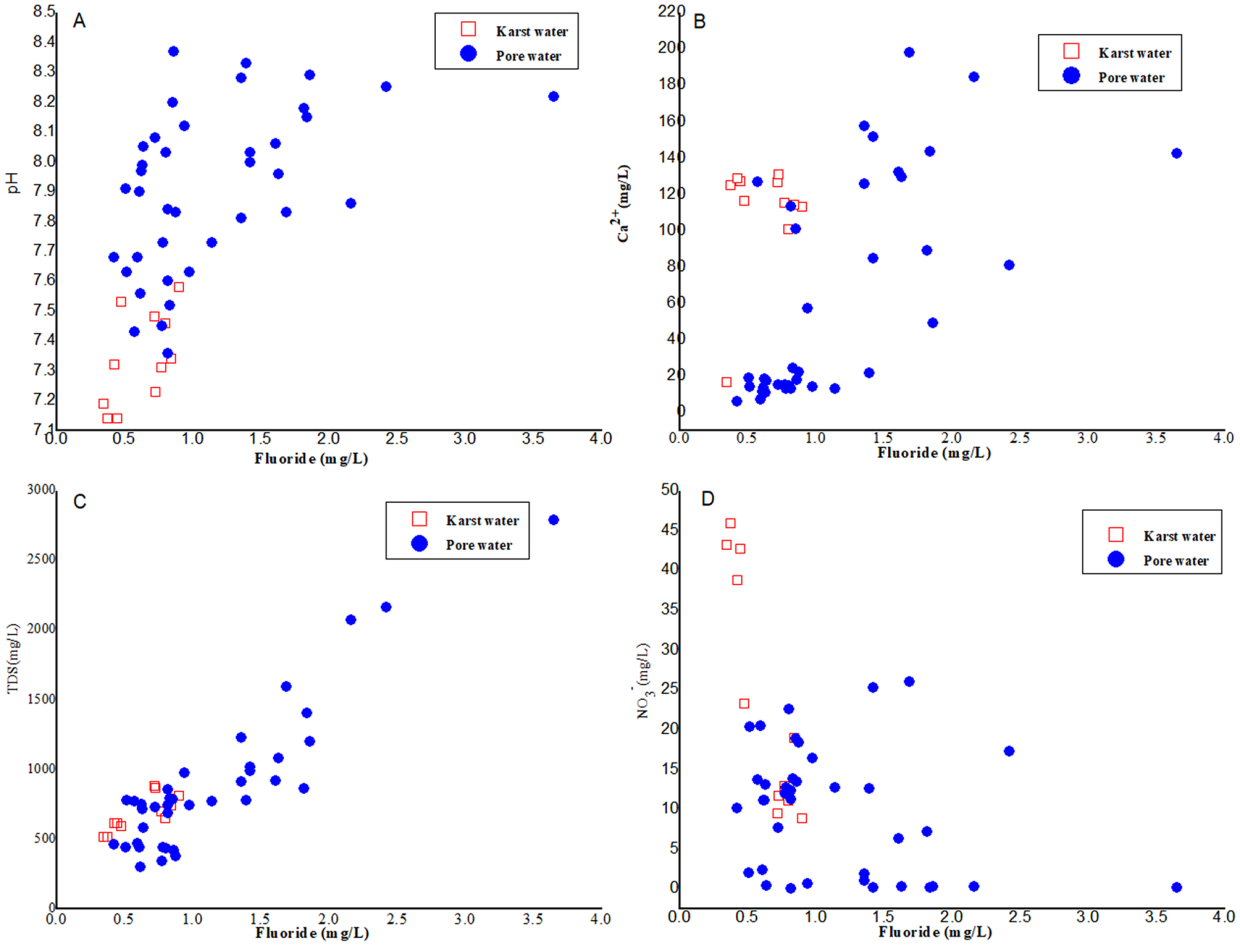
Relationship between the fluoride concentration with major ions in groundwater sources of Huaibei: (**A**) pH, (**B**) Ca^2+^, (**C**) TDS, (**D**) NO_3_^−^.

Correlation coefficients have been shown to be successful in assessing strength and direction-Linear relationship between fluoride and other ions. Many studies shown that pH value plays a decisive role in the occurrence of fluorine in water^9,40^, and alkaline water is more conducive to the dissolution of fluorine-containing minerals. According to Fig. 5A, the positive correlation between pH value and F^−^ in unconsolidated aquifer. The pH value of groundwater plays a decisive role in the occurrence of fluorine. In neutral and alkalescent groundwater, there are many forms of fluorine, such as F^−^, CaF^+^, MgF^+^ et al. With the increasing of pH value, soluble F^−^ occupies the main position. The activity of Ca^2+^ reduces in alkaline and alkalescent groundwater, which weakens the role of F^−^ aggregation ability, and is conducive to the enrichment of F^−^ in groundwater. There is the following equilibrium relationship in groundwater:

3 $$ {\text{Ca}}\left( {{\text{OH}}} \right)_{{2}} { \leftrightharpoons }{\text{Ca}}^{{{2} + }} + {\text{2OH}}^{ - } $$

4 $$ {\text{Ca}}^{{{2} + }} + {\text{ 2F}}^{ - } { \leftrightharpoons }{\text{CaF}}_{{2}} $$

When the groundwater is alkaline or slightly alkaline, the concentration of OH^−^ is higher, and the equilibrium of Eq. (3) moves to the left, resulting in Eq. (4) also moves to the left, so the concentration of F^−^ increases. Alkaline and slightly alkaline water is conducive to the dissolution of fluorinated minerals (CaF_2_), so the concentration of F^−^ increases with the increase of pH value. Therefore, alkaline and alkalescent water is favorable for F^−^ enrichment. The pH value of groundwater in Huaibei Plain is generally between 7.14 and 8.67^28^, which is alkaline and alkalescent, which is favorable for the enrichment of F^−^. Furthermore, F^−^ adsorbed on the clay surface is easily replaced by OH^−^ which leads to the enrichment of F^−^ in alkaline environment. F^−^ can be replaced with hydroxyl (OH^−^) ion and mobilized from micas (such as biotite and muscovite) and clay minerals into groundwater through ion exchange due to the similar ionic radii of OH^−^ and F^−^at alkaline condition^21^.

There is a negative correlation between F^−^ content and Ca^2+^ content (Fig. 5B). The content of fluoride decreases with the increasing of Ca^2+^ content, which may be explained by the interaction between calcium ion and fluorine ion to with the formation of insoluble apatite (Ca_5_(PO_4_)_3_F), fluorite (CaF_2_) and other solid materials^41^. Occurrence of the elevated fluoride concentrations, Ca^2+^ is low obviously due to the fluoride dissolution balance^42^. For the elevated concentration of Ca^2+^ in the centralized water source samples from karst aquifer, F^−^ content is lower than that in decentralized groundwater sources.

According to Fig. 5C, there is a positive correlation between F^−^ content and TDS in water, indicating that F^−^ content in groundwater increases with the increasing of TDS. Evaporation will lead to the gradual concentration of groundwater and the increase of TDS. Minerals with low solubility will reach saturation and precipitate easily. Fluoride in groundwater is mainly comes from water-soluble fluorine ion in the study area, which accumulates continuously with the evaporation of groundwater. The content of TDS increases, the total amount of ions in water increases, which will promote the dissolution of F^−^ into groundwater. The high intensity of annual evaporation from surface water and evapotranspiration from land surfaces are found in the selected area, and the fluoride accumulates with the amount of groundwater, resulting in the higher fluoride content in the shallower well depth.

It has been reported that NO_3_^−^ in groundwater is mainly originated from human activities, For example, coal mines, power plants and other high fluorine waste emissions^28^. NO_3_^−^ contamination in groundwater may be the result of agricultural factors such as fertilizers or animal wastes^42^. The correlation between F^−^ and NO_3_^−^ can reflect the influence of human activities on F^−^ in groundwater. According to Fig. 5D, the poor correlation between F^−^ and NO_3_^−^ in groundwater indicating that the enrichment of F^−^ in groundwater is mainly derived from natural sources.

### Health risk assessment of F exposure in groundwater

**Figure 6:**
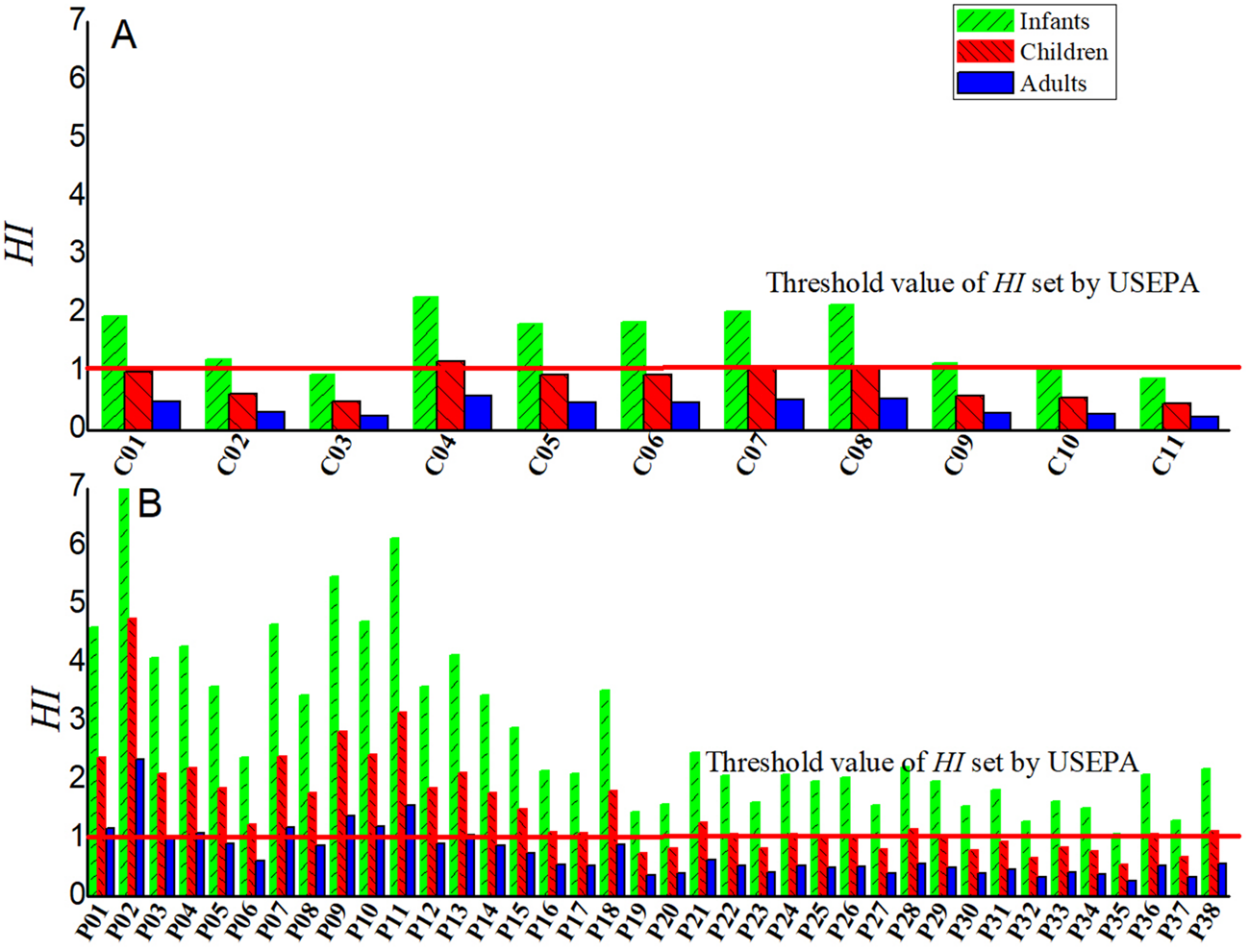
Statistical graph of fluoride noncarcinogenic risk for oral pathway: (**A**) centralized groundwater sources. (**B**) Decentralized groundwater sources.

The *HI* values of fluoride in centralized and decentralized groundwater sources are depicted in Fig. 6. Since the concentration of fluoride in centralized groundwater source is lower than that in decentralized one, the corresponding health risk value is also lower than that in decentralized water source. In centralized groundwater sources, The HI values of fluoride ranged from 0.8872 to 2.2787 (mean 1.5741) and from 0.4563 to 1.1719 (mean 0.8096), from 0.2246 to 0.5769 (mean 0.3946) for infants, children, and adults, respectively indicating that fluoride may cause deleterious health impacts in the order of: infants > children > adults. Whereas for fluoride, *HI* values in 81.82%, 36.36% and 0% of studied locations are above the acceptable USEPA limit of 1 for infants, children, and adults, respectively. In decentralized groundwater sources, *HI* values for infants, children, and adults varied from 1.0646 to 9.2517 (mean 2.8928), from 0.5475 to 4.7580 (mean 1.4877), and from 0.2695 to 2.3424 (mean 0.7324), respectively. *HI* estimated for groundwater in 100%, 71.05% and 23.68% cases are found to be above the safety limit of 1 for infants, children, and adults, respectively. The results means there is a potential risk of developing non cancer health risks in infants, children and adults through drinking water in most locations in the study area. The *HI* values of fluoride for the three exposed groups varied in the same order: infants > children > adults.

Narsimha Adimallaa, b and Hui Qian^43^ investigated assessment of human health risk associated with fluoride contamination in groundwater of Telangana and found children are at highly prone to health risk when compared to adults. In another study, Indrani Mukherjeea and Umesh Kumar Singh^44^ evaluated the health risks to the residents of Birbhum district, India associated with groundwater fluoride exposure. Infants and children are more susceptible to the non-carcinogenic health hazards of fluoride than adults. Similar results are also found in some studies^6,7,45^. It has been reported that infants and children are more sensitive receptors and face higher health risks than adults. For the lower body weight, per unit weight are more sensitive to fluoride pollution for children than that of adults, and long term exposure of groundwater with high fluorine content through ingestion exposure route^46^. The developing infants and children are more vulnerable to fluoride neurotoxicity^15^. It is necessary to carry out groundwater fluoride health risk management to avoid or reduce the impact of groundwater fluoride on the health of infants and children.

It is suggested that for low-risk areas, protection measures should be taken to avoid fluoride pollution in groundwater caused by human activities. For high-risk areas, more efforts should be made to control fluoride in groundwater, and monitoring of dental caries in children should be carried out to prevent the prevalence of dental caries. According to the actual situation, measures should be taken to reduce the fluoride concentration in groundwater, such as precipitation method, adsorption method. Mining deep wells with low fluoride, selecting surface water suitable for drinking to find new water sources, perfecting and improving centralized water supply measures, to ensure the safety of drinking water.

### Uncertainty analysis

Water environmental health risk assessment includes the risk of toxic pollutants to human health through direct contact, ingestion of food, drinking water and respiration. This study only calculated the harm of drinking water intake to human health, without considering other toxic substances and exposure routes. Therefore, the actual health risk level of fluoride pollution in groundwater should be greater than the risk value of this study.

In the health risk assessment of groundwater fluoride, the model parameters are mainly the existing parameters in elder studies, only the influence of fluoride concentration is considered. However, different occupation, gender and consumption habits will lead to differences in health risk assessment parameters. Therefore, it is suggested to carry out basic research on health risk assessment, provide parameters suitable for the health risk assessment of population in the study area, improve the accuracy of groundwater health risk assessment, and provide scientific basis for the safety management of regional groundwater quality. This study on the health risk of fluoride in groundwater in Huaibei is preliminary and will be further improved in the future work.

## Conclusions

The concentration of fluoride selected from centralized groundwater sources and decentralized groundwater sources are ranged from 0.35 to 0.90 mg/L and 0.42 to 3.65 mg/L, with an average of 0.62 and 1.42, respectively. The average concentration of fluoride in the decentralized groundwater source is higher than that of centralized groundwater sources. And the vertical distribution of fluoride in groundwater tend to decreases with the increase of well depth. Groundwater fluoride mainly derived from fluoride-rich minerals by the effect of chemical weathering, rock–water interaction, ion-exchange and evapotranspiration. High fluoride concentrations are associated with weakly alkaline conditions, moderate total dissolved solids. Among them, calcium ions are regarded as the dominant ions for association of fluoride in groundwater. The distribution attributed to different geological conditions, and also related to environmental conditions and high fluoride waste discharge.

In centralized groundwater sources, The *HI* values of fluoride ranged from 0.8872 to 2.2787 (mean 1.5741) and from 0.4563 to 1.1719 (mean 0.8096), from 0.2246 to 0.5769 (mean 0.3946) for infants, children, and adults, respectively. While in centralized and decentralized groundwater sources, *HI* values for infants, children, and adults varied from 1.0646 to 9.2517 (mean 2.8928), from 0.5475 to 4.7580 (mean 1.4877), and from 0.2695 to 2.3424 (mean 0.7324), respectively. Results indicated that the *HI* values for studied groups are in the same order: infants > children > adults. The infants and children are more prone to risk of fluoride in the groundwater compared with adults. Measures should be taken to ensure the safety of drinking water from fluoride contamination.
